# Adherence to Remote Microphone System use at school in children and adolescents with hearing loss

**DOI:** 10.1590/2317-1782/20212020326

**Published:** 2022-01-12

**Authors:** Giovana Targino Esturaro, Bruna Capalbo Youssef, Luisa Barzaghi Ficker, Tatiana Medeiros Deperon, Beatriz de Castro Andrade Mendes, Beatriz Cavalcanti de Albuquerque Caiuby Novaes

**Affiliations:** 1 Programa de Pós-graduação em Fonoaudiologia, Faculdade Pontifícia Universidade Católica de São Paulo – PUC-SP - São Paulo (SP), Brasil.

**Keywords:** Hearing, Hearing Aids, Wireless Technology, Schooling, Parents, Hearing Loss, Teenager

## Abstract

**Purpose:**

To identify relationships between Remote Microphone System (RMS) use in the classroom and the schools’ and teachers’ characteristics.

**Methods:**

We analyzed 120 subjects aged 5 to 17 years with hearing loss who had received an RMS from a health service accredited by the Unified Health System (SUS). The teachers of RMS users were the other subjects in the study. We analyzed the patients’ medical records and interviewed their parents/guardians at the follow-up visit to verify issues related to the RMS and its use at school. We contacted the schools over the phone and visited some of them.

**Results:**

Of the students, 54% used the device at school; 22% involuntarily did not use it; and 24% voluntarily did not use it. The Speech Intelligibility Index pattern of those who used the RMS was similar to those who involuntarily did not use it. There was a significant difference between the type of school and educational level – 86% of regular school students and elementary school students tend to use the device more often (62%).

**Conclusion:**

Most subjects use the RMS at school. The students’ educational level also interfered with the adherence to RMS use, as elementary school students had a higher adherence. The data suggest that the coordination between health services and schools favors RMS use. However, when the parents mediate this relationship, other factors interfere with the systematic RMS use in the school routine.

## INTRODUCTION

The hearing is responsible for picking up stimuli and has an essential role in the overall development of children. One of the requirements for children to acquire oral language is their ability to detect, localize, discriminate, memorize, recognize, and understand sounds^(1,2)^.

All the environments to which the child belongs must be favorable to pragmatics, language, and hearing, enabling not only the communicative interaction but also the best use of situations that involve dialogue to favor their development. The school, for instance, is one of such environments. Since it is a place where the child spends much of their time, it must potentialize its students’ development^(3)^.

In addition to favorable environments, the hearing aid (HA) must be programmed and verified to ensure that children with hearing loss can hear speech sounds, which is necessary for their language development^(4)^.

The Speech Intelligibility Index (SII) is an objective measure taken in the process of verifying the HA. It informs the amount of speech information the person hears with and without amplification and is one of the manners to quantify the relationship between the speech signal and the speech recognition results^(5)^.

The capacity to process and understand the teachers’ speech is essential to their school learning. Studies demonstrate that, at school, children spend 45% of the time involved in activities in which the teacher’s and classmates’ speech is predominant. Hence, having adequate access to the teachers’ voice is essential to their school learning^(6,7)^. However, adherence to the device only occurs when all the people involved with its use (health professionals, family, and educators) work in partnership to ensure its effective and proper use^(8-10)^.

The remote microphone systems (RMS) is an assistive technology that provides technical help to people with hearing loss. It includes products, instruments, equipment, and technology adapted or specially projected to improve the functioning of people with a disability, aiding them to achieve total or assisted personal autonomy (Law no. 5296, of December 2, 2004), providing direct access to the interlocutor’s voice.

In this context, the RMS is a wireless technology that picks up the interlocutor’s voice (teacher, therapist, or parents) with a microphone connected to a transmitter, which in turn sends the speech signal to a receiver connected to the user’s HA or cochlear implant (CI). Hence, it minimizes problems related to distance, noise, and reverberation, improving the signal-to-noise ratio in different environments.

The evolution of frequency modulation (FM) technology for digital transmitters furnished further benefits to the users, as it enables better speech recognition in noise and diminishes interferences, making sound transmission more constant^(11)^. We chose to use the term remote microphone because it encompasses all such technologies.

In 2013, Regulation no. 1.274/GM/MS, promulgated by the Ministry of Health, included the FM system^(12)^ in the table of procedures, medications, ortheses, prostheses, and special material from the Unified Health System (SUS, in Portuguese). The purpose was to implement new initiatives and intensify the measures already being taken by the government to benefit people with a disability and improve their access to basic rights, such as education. Thus, HA and/or CI users aged 5 to 17 years – particularly those with speech recognition abilities – could turn to SUS to obtain this resource, making their learning at school easier.

In 2020, the SUS began an analysis to extend RMS to people of any age and at any educational level with hearing loss – SUS, Regulation no. 3, of February 19, 2020^(13,28)^.

Schools are excessively noisy environments, and noise interferes with the student's academic performance, especially those with hearing loss, with a direct impact on their listening effort^(10,14)^. The reverberation in the classrooms poses difficulties in understanding the message conveyed by the interlocutor, requiring more energy for the person to understand the content that is being said. The best way to shorten the distance between the speaker and interlocutor is with assistive technology, using RMS to improve the signal-to-noise ratio in the calssroom^(8,15,16)^.

The effectiveness and success of RMS use depend on various factors, including the partnership between health and education. They need to work together in this process to benefit the student. In the case of children with hearing loss and oral communication, they must be ensured access to speech perception and pedagogical content^(17,18)^. However, some difficulties appear in this relationship, such as excessive bureaucracy and lack of human resources and time, which makes the school-health partnership difficult^(19,20)^.

Concerning educational level, it has been demonstrated that elementary school students use RMS more than middle and high school students. This same study observed that subjects who did not voluntarily use the equipment had good audibility (above 60%) in silent situations^(21)^.

A study that analyzed 185 medical records of adolescents who had been fitted with RMS in 2013 to 2016 reports that some of the subjects who did not use the device were ashamed to use it (40%), the HA or RMS of others was not working (20%), the teacher did not encourage them or the students did not feel the need to use it (27%), or had difficulties handling the HA and rejected it (10%)^(22)^.

The hearing health service or specialized rehabilitation center must quickly provide the necessary support to solve problems and help the school get adapted to the device, besides properly adjusting the RMS to the child’s HA and/or CI. Thus, the objective of this paper was to identify relationships between systematic RMS use in the classroom and the students’, schools’, and teachers’ characteristics.

## METHODS

This is an exploratory qualitative and quantitative study with teachers who worked with students with hearing loss who used RMS in the school routine. This study was submitted to the Research Ethics Committee of the *Pontifícia Universidade Católica* (Pontifical Catholic University) of São Paulo and *Plataforma Brasil* and was approved under evaluation report number 1.110.125 (CAEE: 45415514.1.0000.5482). All the subjects’ parents/guardians signed the informed consent form.

We analyzed 120 subjects with mild to profound sensorineural hearing loss who had been fitted with RMS and undergo audiological follow-up at a health service accredited by the SUS as a specialized rehabilitation center II – Auditory and Intellectual (CER II), in the city of São Paulo between January and December 2017. The subject characterization is shown in Table 1. The other subjects of this study were teachers of the RMS users. The research inclusion criteria are based on those described in the FM Regulation 1.274.

**Table 1:** Subject characterization

Characterization of subjects					
**Sex**		Femaly	Male		
	N	59	61		
	%	49%	51%		
**Socioeconomic Level**		A+B1	B2+C1	C2+D+E	
	N	8	67	45	
	%	7%	56%	38%	
**Educacional Level**		Fundamental I	Fundamental II	Ensino Médio	
	N	68	41	11	
	%	57%	34%	9%	
**Degree of Hearing loss**		Normal/Mild	Moderate	Severe	Profound
	OD	21	38	35	26
	OE	12	40	36	32

Legend: OD- right ear; OE- left ear

Before the assessment, we analyzed the medical records to obtain information such as sex, age, auditory thresholds at 500 Hz to 4 kHz in both ears, SII values at 65 dB in the best ear, place of residence, the student’s educational level (elementary, middle, or high school, or adult education), socioeconomic classification, parents’/guardians’ educational level, and type of school the student attended (regular, regular with an interpreter, or special school taught in Brazilian Sign Language [LIBRAS]).

In the annual or bi-annual follow-up visit to the institution to undergo further audiological examinations and verify the HA, we carried out the following procedures:

1. Interview with the parents/guardians to verify issues related to the RMS and its use at school;
2. Administration of a questionnaire on the families’ socioeconomic classificaiton^(23)^;
3. Verification of the HA and the mean daily hours using it;
4. Verification of RMS functioning;
5. Classification regarding RMS use:

Yes – using RMS

- If they use it regularly at school (more than 1 and a half hours a day at school)

Not using RMS – involuntarily

- RMS is being repaired;
- RMS was lost or stolen;
- The teacher does not want to use it at school.

Not using RMS voluntarily

- Returned the RMS;
- Attends special school (LIBRAS);
- The student does not want to use the RMS at school.

1. Material handed to the parents/guardians for them to take to the school’s education staff, containing instructions on the proper use of the equipment; the Brazilian SIFTER – Screening Instrument for Targeting Educational Risk in middle and high school students; research presentation letter; and informed consent form.

### Phone contact with all the schools

Besides the material sent through the families, we contacted some schools over the phone and visited them. It is part of the institution’s protocol to visit the schools to explain and guide about the RMS, even when the parents do not ask us to go to the school. We gave priority to those which the parents or children asked us to visit or when the school invited us to go.

All the schools are in the city of São Paulo. We found their address and principal with the following procedures:

1. Searching on Google for the name of the school to find their phone number and address, or using information brought by the parents;
2. Contacting the schools of the students in this research over the phone to talk with the teacher, coordinator, support room teacher, principal assistant, and/or interpreter responsible for the student; those who were available at the moment were invited to visit the school;
3. Scheduling the visit according to the school’s availability. In case the RMS was not working or waiting for compatible receivers (involuntary non-use), we did not visit the school.

### School visit

- Visit paid to the student’s school scheduled over the phone with the available professional responsible for the student.

### School visit plan

1. Present the RMS to the education staff who were in the meeting to explain the benefits of using it and how to handle the device;
2. Show a video demonstrating the benefits of the equipment and the difference in the child’s hearing with HA and HA+RMS, and how to care for the equipment;
3. Demonstrate the RMS with an earphone for the teachers/coordinators to have a real sensation of using the RMS, so they could relate to how the students hear in the classroom;
4. If the child was at the school and could pause their activities, we asked to visit their classroom to demonstrate the RMS in use;
5. Hand the explanatory booklet (Annex 1) to the school, as well as the Brazilian SIFTER^(24)^.

After the school visit, we scheduled a new appointment with the families (unless they already had a follow-up visit scheduled) to give feedback on the visit to the school and new instructions.

### Data analysis

We analyzed the relationships between the audiological and demographic characteristics of the subjects and schools, relating them to the adherence to RMS use. We also performed a qualitative analysis based on the school visits, which we had paid to guide them and obtain reports regarding difficulties and/or benefits of the student’s RMS use.

The data were entered into a spreadsheet and analyzed with the Statistical Package for the Social Sciences (SPSS), version 22.0 for Windows. We used nonparametric tests to compare the qualitative variables, specifically, the Kruskal-Wallis test to compare two or more categories. If there was a significant difference, we used the Mann-Whitney U test for paired comparisons between the groups.

## RESULTS

A total of 120 subjects fitted with RMS attended the follow-up visit at the institution in the year we collected the data. Of these, 54% (n=65) were females and 46% (n=55), males. As for the socioeconomic classification, 63% (n=76) were from class C, 28% (n=34) from class B, 8% (n=9) from class D, and one family (1%) from class A.

We found that 54% (n=65) used the device at school; 22% (n=26) involuntarily did not use it (the HA or RMS had been lost or stolen, or was being repaired; the HA was incompatible with the receiver; and/or the teachers did not want to use it); 24% (n=29) voluntarily did not use it (they returned the device saying they did not see any benefit in it; oral language was not their main form of communication; LIBRAS was their main form of communication and they attended a special school, where the teachers taught in this language; and/or the student did not want to use the device at school).

**Figure 1:**
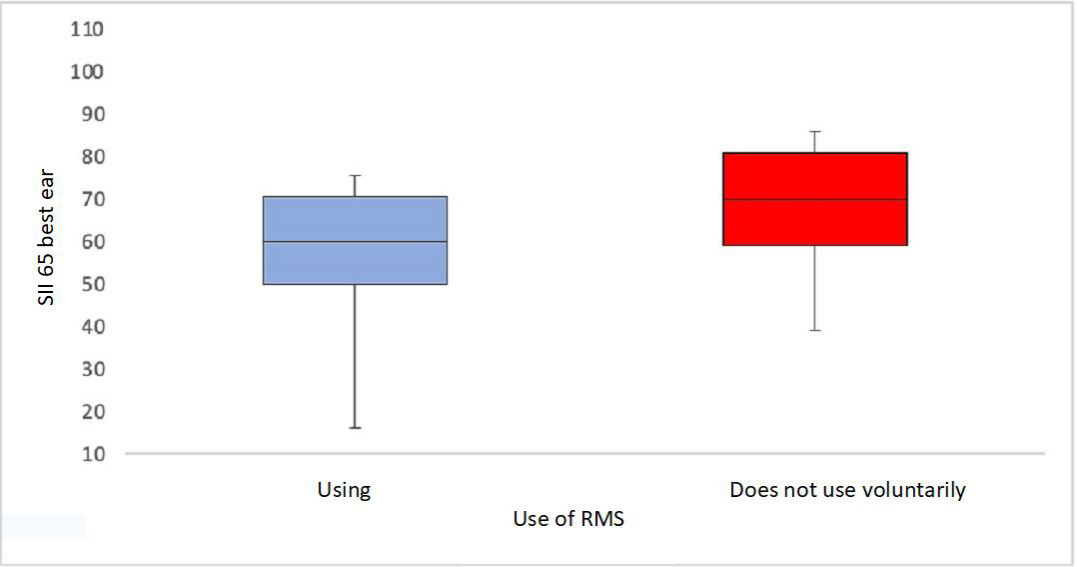
Box plots of subject distribution regarding Remote Microphone use (using it and kept from using it – grouped in one – and not using it) in relation to the Speech Intelligibility Index (n=120)

We used the SII to establish the degree of hearing loss. The distribution of RMS use and the subjects’ SII is shown in Figure 1, in which we see a great SII variability at 65 dB in subjects who use and involuntarily do not use the device. In those who voluntarily do not use the device, there is a trend to a higher SII value. This difference was significant (p < 0.05).

**Figure 2:**
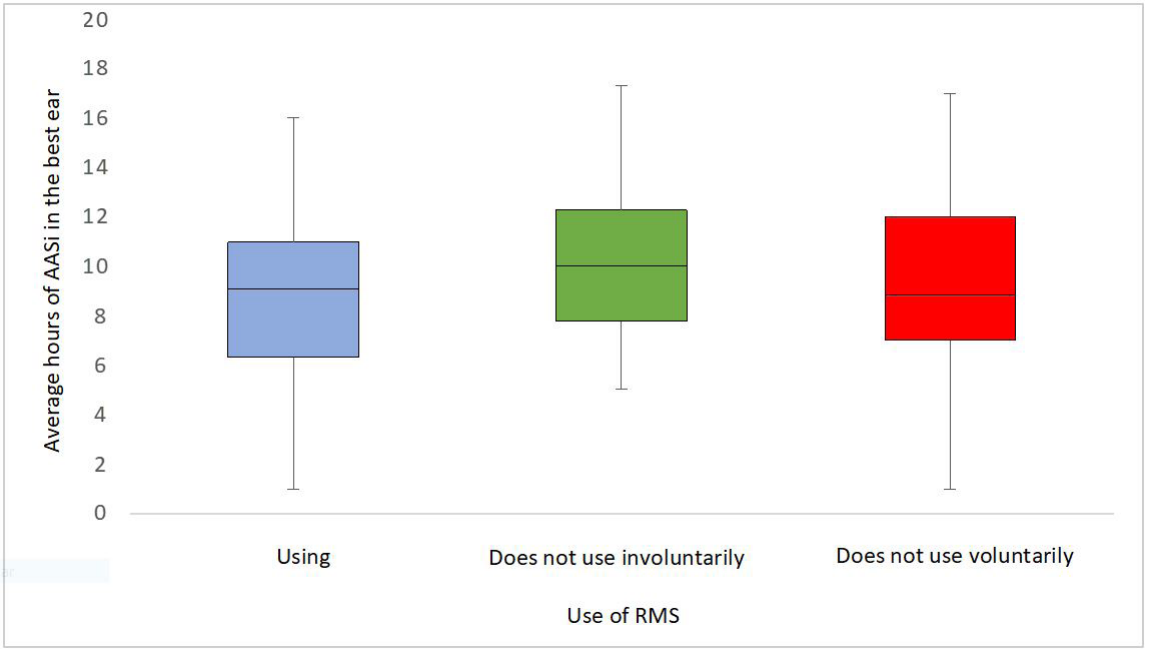
Box plots of the median daily use of the hearing aid in each category of microphone use

The lowest mean and median HA daily use were observed in those who voluntarily did not use the device. The distributions of this variable are shown in Figure 2.

Concerning the type of school, 86% (n=103) attended regular schools, 7% (n=8) attended regular schools with a LIBRAS interpreter, and 7% (n=9) attended special schools, exclusively taught in LIBRAS. Of the 120 subjects, 57% (n=68) were enrolled in elementary school, 34% (n=41) in middle school, and 9% (n=11) in high school.

We compared their educational level with RMS use and found high significance levels. The comparison between the three educational levels had a significance of p=0.028, while that between elementary and high school had a significance of p=0.008.

**Figure 3:**
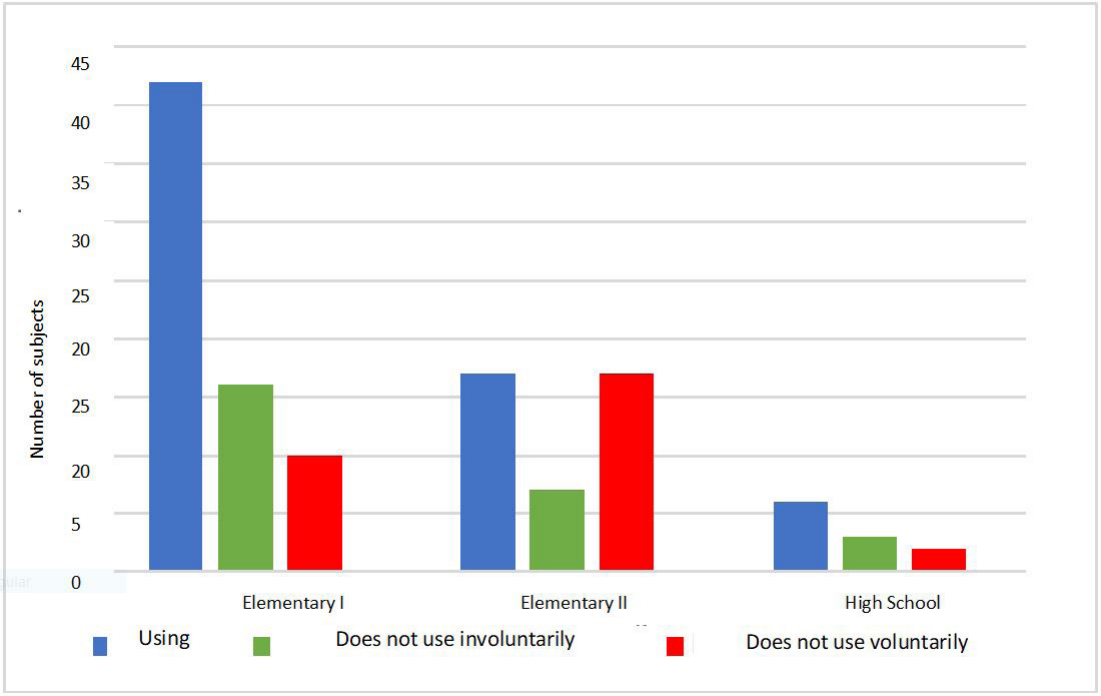
Subject distribution relating the Remote Microphone use to the student’s educational level (n=120)

The educational level comparative analysis revealed that 62% of the subjects in elementary school used the RMS. This was the educational level with the highest adherence to device use, followed by high school (55%) and middle school (41%). We must point out that there were only 11 subjects in high school, which makes comparison difficult (Figure 3).

The distribution of RMS use by elementary (n=68) and middle school subjects (n=41), when compared with one another, have some differences in the number of subjects in each category we analyzed, as shown below.

There were more subjects in elementary than in middle school using (difference in n=25) and involuntarily not using the device (difference in n=9).

Middle school surpasses elementary school only in the number of subjects who voluntarily did not use the device (difference in n=7).

**Figure 4:**
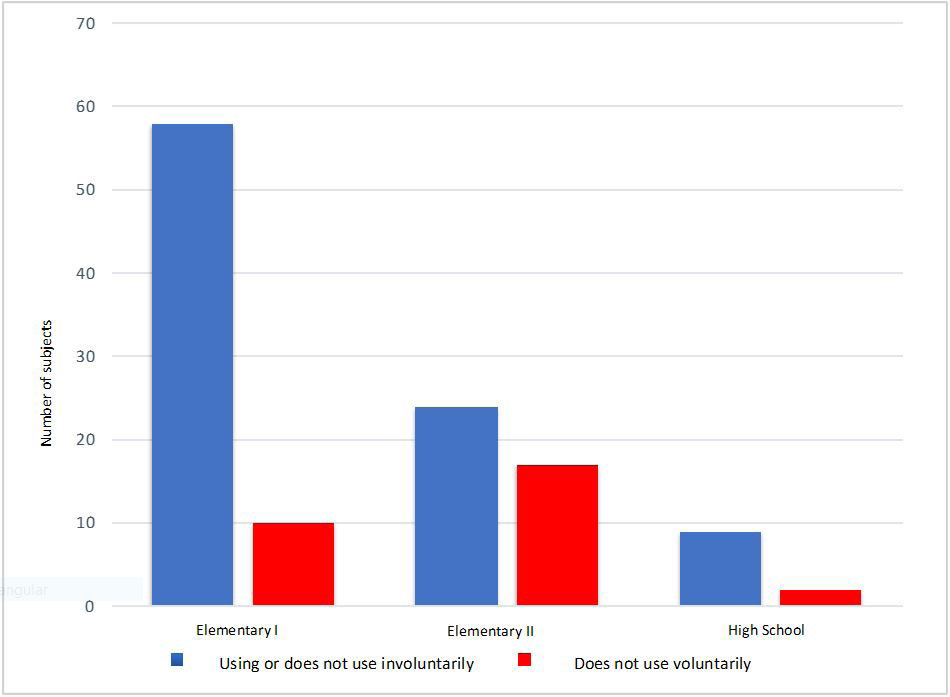
Student distribution relating the Remote Microphone use (using it and kept from using it – grouped in one – and not using it) in relation to the student’s educational level (n=120)

From another perspective, the subjects who were using the RMS at school were grouped with those who were not using it due to factors beyond their control (involuntarily not using the device). This distribution makes more evident the greater difficulty adhering to its use on the part of middle school students (Figure 3 and Figure 4).

One of the reasons to contact the school was to give the Brazilian SIFTER, either through the families or in the visit, to the education staff that met with the health professionals. We received back only six (5%) out of the 120 questionnaires we had delivered. Given the low adherence to it, we could not analyze the answers to this instrument.

**Figure 5:**
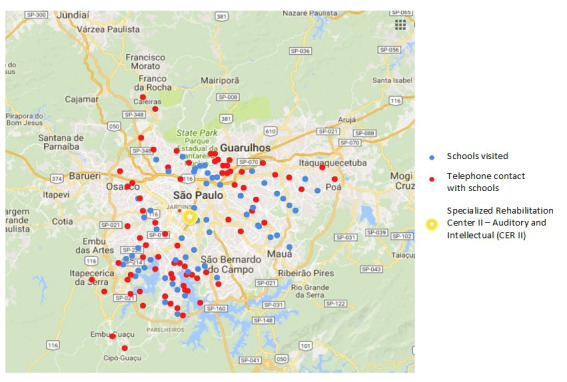
Subject distribution relating the schools visited and telephone contacts that were made (n=120)

In a qualitative approach to improve the process of adapting to the device at schools and meet the parents’ needs, we contacted the schools over the phone and scheduled visits to 28 of them, as shown in the map below (Figure 5).

We scheduled 14 visits based on requests of the users’ families to better guide their education staff, and another 14 visits based on the institution’s protocol, as long as the schools agreed with it.

In 75% (n=21) of the schools we visited, the children were using the RMS in the classroom; in 14% (n=4), the student “did not get adapted to using the RMS”, according to the school; in 7% (n=2), the students’ HA/RMS were being repaired; and one (3%) was a special school, taught in LIBRAS. Their availability to receive the health service team for guidance was probably a determining factor to this distribution. The schools whose students used the device regularly seemed to be more interested in receiving guidance.

In 54% (n=15) of the schools we visited, teachers and coordinators participated in the meeting; in 29% (n=8), only the coordinator participated, who committed to passing on to the teachers at another moment what had been discussed during the visit; in 21% (n=6), only the teachers participated.

Considering that the students were using the RMS in the classroom in 23 out of the 28 schools that agreed to have us visit them, the coordinators were present in 91% of the visits, and both the coordinator and teachers were present in 56% of them.

## DISCUSSION

A total of 120 subjects fitted with RMS in 2017 participated in the study. They were part of the Agreement Term, a project involving the specialized rehabilitation center – hearing loss and the Municipal Government of São Paulo, via the Department of Participation and Partnership/Municipal Council of Children’s and Adolescents’ Rights.

The adherence to RMS is much more complex than simply handing the device and expect the subject to be successful in using it, with no coordination between family, teachers, and health professionals^(17,18)^.

We observed that 54% (n=65) of the students were using the device at school, 22% (n=26) were involuntarily not using it, and 24% (n=29) were voluntarily not using it. According to the authors^(10,20)^, this may be related to various factors, including lack of information on the part of the teacher about how to use the equipment and about the student’s hearing loss. In other cases, the student refused to use the device, claiming they were ashamed to do it in front of their classmates.

In this study, we related the equipment use to the SII. The subjects who used the device had a similar SII pattern to those who involuntarily did not use it, whereas those who voluntarily did not use it tended to have a higher SII. This can be explained by the students’ not feeling differences at first with and without the RMS and the family’s not understanding the real need for the device and not insisting on its use^(10,21)^. The subjects who voluntarily did not use the equipment had good audibility (above 60%) in silent situations, which may explain the low adherence to the RMS in the initial adaptation, as they claim they hear well with the HA or CI alone^(21)^.

The regulation of the Ministry of Health does not provide for replacements in case the equipment is lost, stolen, or defective. In this study sample, 22% of the subjects involuntarily did not use the device because it had been lost or stolen, the HA or RMS was being repaired, the HA was incompatible with the RMS receiver, and/or the teacher refused to use it – which agrees with the literature. In another study with the population who had received the RMS between 2013 and 2016, the equipment of 16% of them was not working^(22)^.

Many subjects stopped using the RMS for factors beyond their control, being deprived of its benefits. When RMS users are grouped with those who would use it if they were not prevented from it, this difference further highlights the increase in the number of potential RMS users at school. They would not be kept from using the RMS if replacement policies were carried out, as stated in Regulation 1.274. Also, Report no. 58 of the National Commission for Incorporating Technologies into SUS (CONITEC, in Portuguese) points out the impact on the budget when this assistive technology is incorporated, considering the target audience originally subject to this type of intervention. Having the RMS systems available at SUS is a great improvement towards the rehabilitation of children with hearing loss. Nonetheless, the distribution is still short of the recommended by the Ministry of Health, as it is unequal between the regions of the country and the device availability has decreased in recent years^(25)^. Some studies^(25,26)^ argue that these technologies have a 4-year service life and need to be replaced from time to time. However, two years after the number of concessions were expected to double, as mentioned above, no FM system had been replaced, neither had the indicated technology been revised.

The student’s educational level was also a factor that interfered with the adherence to RMS use – 57% (n=68) of the subjects were attending elementary school, and they were the ones who most used the RMS (62%). This agrees with the statistical tests, with a significance level of p=0.028 in the comparison between educational levels (elementary, middle, and high school) and device use. We observed a greater use in elementary school students, even when grouped with those who involuntarily did not use the equipment. Likewise, when the educational level (elementary and high school) was compared with RMS use, we also found a significance of p=0.008. This difference suggests that having only one teacher in the classroom in elementary school helps the students’ use of the electronic device, the guidance, and the adherence to its use in the school routine – which was observed by the author, as well^(21)^.

The literature shows that the coordination between health care and school is still challenging^(19)^. In this study, we verified that contacting the school was not an easy process. We had to call them over the phone many times before being successful, and the professionals had to go long distances to visit the schools, which also interferes with the adherence^(20,27)^.

This is further evidenced by the low adherence to the questionnaire, as only 5% of the teachers answered the SIFTER, mostly claiming they lacked the time to fill in and send the questionnaire. According to the author^(19)^, such a lack of time was a factor that also hindered the cooperation between health care and education.

We analyzed the children who did not use the RMS and related them to the participants in the meetings when we visited the schools. We identified that those who participated were the support room teacher and the specialized educational support teacher. These professionals spend only a few hours a week with the children; hence, the absence of the main teacher and education coordinator in these meetings may negatively interfere with RMS use.

On the other hand, the situation was different when we analyzed the subjects who used the RMS. In most of them (56%), the teachers and coordinators participated in the meeting. We concluded that having the whole team together in these meetings helps them adhere to RMS use, as it does not depend on the students alone, but collectively on the school, family, and health system.

Successful RMS use requires coordination between health, family, and education. We must consider, though, that the relationship between the health service and the school does not seem to be systematic and depends on informal contact between these teams. Seemingly, the regulation of the Ministry of Health needs to be revised to include the replacement of devices due to loss, theft, and receiver incompatibility when the HA is replaced, and the maintenance of defective devices.

Further research on this topic is essential to broaden the awareness of verbal child inclusion in school settings and develop more protocols to help their adaptation to RM at school. Furthermore, the partnership between health and education must be strengthened to benefit the children with hearing loss.

## CONCLUSION

We concluded that:

● The majority of the subjects used the RMS at school.

● Loss, theft, and defect were some of the reasons for involuntarily not using the device.

● The student’s educational level was also a factor that interfered with their adherence to RMS use; students attending elementary school were the ones who most used the RMS.

● Difficulties reaching the teacher contributed to the non-adherence to device use, as it was not always possible to give the necessary instructions regarding its consistent and adequate use.

## Supplementary material

Supplementary material accompanies this paper.

**Annex 1:** Sounds and my hearing

This material is available as part of the online article from https://www.scielo.br/j/codas

## References

[1] Bonaldi LV, Boéchat EM, Menezes PL, Couto CM, Frizzo ACL, Scharlach RC, Anastasio ART (2015). Tratado de audiologia..

[2] Butugan O, Santoro PP, Almeida ER, Silveira JAM, Grasel SS (2000). Diagnóstico precoce da deficiência auditiva no primeiro ano de vida de crianças com alto risco através de audiometria de tronco cerebral. Pediatria..

[3] Delgado-Pinheiro EMC, Antonio FL, Libardi AL, Seno MP (2009). Programa de acompanhamento fonoaudiológico de professores de alunos deficientes auditivos que utilizam a comunicação oral. Rev Distúrb Comun..

[4] McCreery RW, Walker EA, Spratford M, Bentler R, Holte L, Roush P (2015). Longitudinal predictors of aided speech audibility in infants and children. Ear Hear.

[5] Figueiredo RSL, Mendes B, Cavanaugh MCV, Novaes B (2016). Classificação de perdas auditivas por grau e configuração e relações com Índice de Inteligibilidade de Fala (SII) amplificado. CoDAS.

[6] Lemos ICC, Jacob RTS, Gejão MG, Bevilacqua MC, Feniman MR, Ferrari DV (2009). Sistema de freqüência modulada no transtorno do processamento auditivo: prática baseada em evidências. Pró-Fono R Atual Cient..

[7] Boothroyd A, Smaldino J, Flexer C (2012). Handbook of acoustic accessibility..

[8] Anderson KL, Goldstein H (2004). Speech perception benefits of FM and infrared devices to children with hearing aids in a typical classroom. Lang Speech Hear Serv Sch.

[9] Bertachini ALL, Pupo AC, Morettin M, Martinez MAN, Bevilacqua MC, Moret AML (2015). Sistema de Frequência Modulada e percepção da fala em sala de aula: revisão sistemática da literatura. CoDAS.

[10] Thibodeau L (2010). Benefits of adaptive FM systems on speech recognition in noise for listeners who use hearing aids. Am J Audiol.

[11] Wolfe J, Morais M, Schafer E, Mills E, Mülder HE, Goldbeck F (2013). Evaluation of speech recognition of cochlear implant recipients using a personal digital adaptive radio frequency system. J Am Acad Audiol.

[12] Brasil (2013). Portaria nº 1.274, de 25 de junho de 2013.

[13] Brasil (2020). Portaria nº 3, de 19 de fevereiro de 2020.

[14] Benítez-Barrera CR, Thompson EC, Angley GP, Woynaroski T, Tharpe AM (2019). Remote microphone system use at home: impact on child-directed speech. J Speech Lang Hear Res.

[15] Dreossi RCF, Momensohn-Santos T (2005). O ruído e sua interferência sobre estudantes em uma sala de aula: revisão de literatura. Pró-Fono R Atual Cient.

[16] Cruz AD (2018). Esforço auditivo e fadiga em adolescentes com deficiência auditiva - uso do sistema FM.

[17] Lewis MS, Hutter M, Lilly DJ, Bourdette D, Saunders J, Fausti SA (2006). Frequency-modulation (FM) technology as a method for improving speech perception in noise for individuals with multiple sclerosis. J Am Acad Audiol.

[18] Campos NB, Delgado-Pinheiro EMC (2014). Análise do ruído e intervenção fonoaudiológica em ambiente escolar: rede privada e pública de ensino regular. Rev CEFAC.

[19] Penso MA, Brasil KCTR, Arrais AR, Lordello SR (2013). A relação entre saúde e escola: percepções dos profissionais que trabalham com adolescentes na atenção primária à saúde no Distrito Federal. Saude Soc.

[20] Miguel JHS, Novaes BCAC (2013). Reabilitação auditiva na criança: adesão ao tratamento e ao uso do aparelho de amplificação sonora individual. ACR.

[21] Esturaro GT, Novaes BCAC, Deperon TM, Martinez MAN, Mendes BCA (2016). Uso de sistema de transmissão sem fio e desempenho de estudantes com deficiência auditiva na perspectiva de professores. Distúrb Comun.

[22] Sposito C (2017). Resistência ao uso do sistema FM por adolescentes em um serviço público de saúde auditiva: fato ou mito?.

[23] ABEP: Associação Brasileira de Empresas de Pesquisa (2018). Classificação Socioeconômica - Critério de Classificação Econômica Brasil..

[24] Granço FS (2010). Adaptação cultural do questionário: screening instrument for targeting educational risk in secondary students (S.I.F.T.E.R).

[25] Dutra MRP, Ferreira MAF (2021). Provision of the frequency modulation system for the hearing impaired. Rev Bras Otorrinolaringol.

[26] Silva EJ, Carneiro LA, Jacob RTS (2020). O Poder Judiciário e o acesso ao Sistema de Frequência Modulada: uma análise sobre a efetivação das políticas públicas em saúde auditiva. Audiol Commun Res.

[27] Youssef BC, Mendes BDCA, Costa EDC, Ficker LB, Novaes BCAC (2017). Efetividade na adesão a reabilitação auditiva em crianças: Grupo de Adesão Familiar e terapia inicial. Distúrb Comun.

[28] Silva EJ, Carneiro LA, Jacob RTS (2020). The Judiciary and access to the Frequency Modulation System: an analysis of the effectiveness of public policies on hearing health. Audiol Commun Res.

