# Supplementary material for: Adherence to Remote Microphone System use at school in children and adolescents with hearing loss
Source: Codas. 2022 Jan 12;34(3):e20200326. doi: 10.1590/2317-1782/20212020326 (PMC9769424; doi:10.1590/2317-1782/20212020326)
Supplement: Supplementary file 1 [file codas-34-3-e20200326-suppl1.pdf]

FM Project

Derdic | PUC-SP

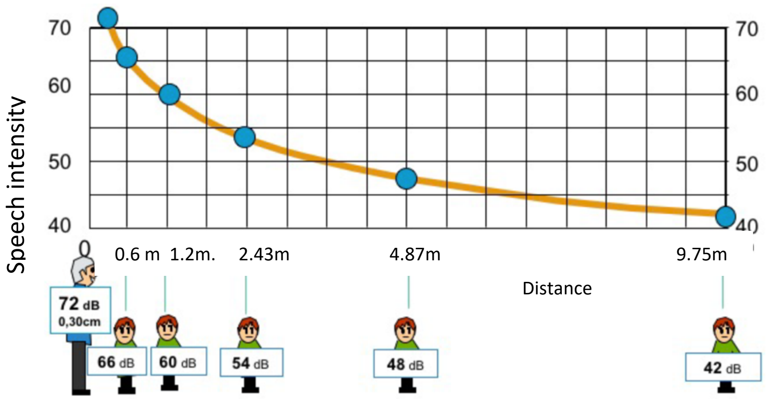

MY NAME

School:

Sounds and my hearing

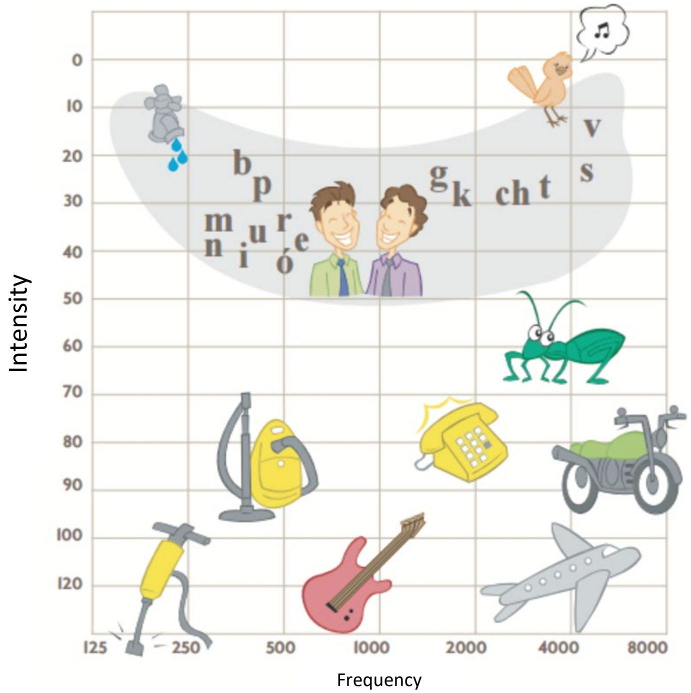

My hearing loss

Type of hearing loss

Right ear:

Left ear:

Degree of hearing loss

Right ear:

Left ear:

Sounds and my hearing

1 - Hearing aid (HA)

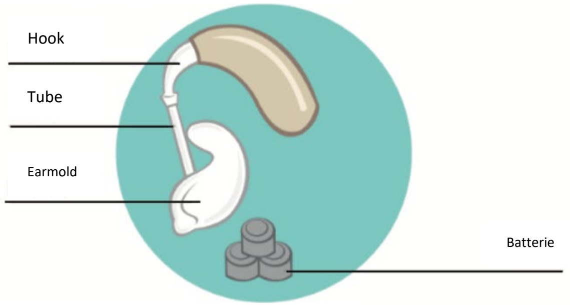

2 - Cochlear implant

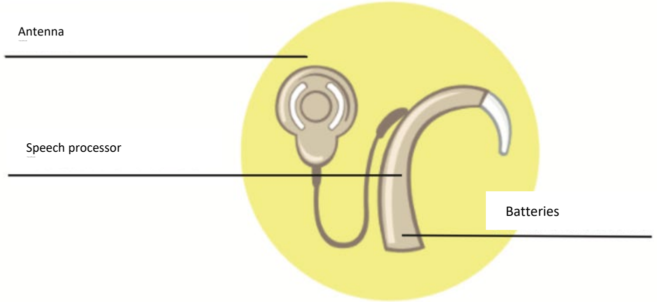

Notes

HA/CI model
Right ear:
Left ear:

What my FM does and how it works

The FM system is an educational tool developed for children with hearing loss who use hearing aid and/or cochlear implant to help them understand what their teachers, parents, and therapists say in noisy environments, shortening the distance between the speaker and the listener, especially at school.

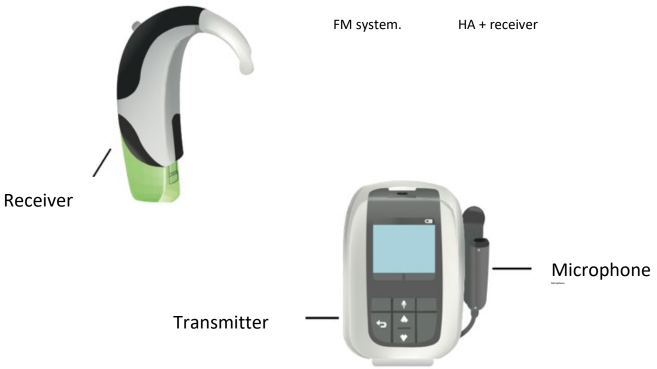

Notes

HA/CI model
Right ear:
Left ear:

Effects of Reverberation and distance to the teacher

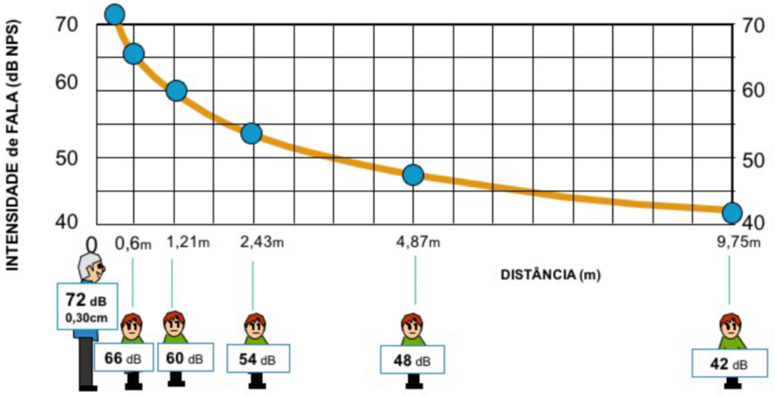

We must think of all the places where a child belongs to as favorable to developing language and hearing – places that enable communicative interaction. The school is one of these places; that's where the child spends much of their time (Delgado-Pinheiro, 2009).

Students spend 45% of their time at school involved in activities in which the teacher's and classmates' voices predominate. Therefore, we can easily conclude that both the message and the ability to auditorily process it are essential to school learning.

Children with hearing loss need to make a greater effort than their hearing peers – especially at school, regardless of the technological resource they use (HA or CI) – to understand messages conveyed to them (Hicks and Tharpe, 2002).

Some tips to use the FM well

→ The microphone must be attached to the clothes, at least nine inches below the mouth to avoid amplifying noises from breathing, the mouth, and accessories (necklaces, scarf, and buttons).
→ Avoid hitting or drooping the device.
→ When it gets dirty with chalk dust, clean it with a tissue or dry cloth.
→ Don't let it get wet.
→ Avoid humidity because it can oxidate.
→ If it malfunctions, check with the family if its batteries have been properly replaced.

Frequently asked questions

1. **Is it enough to use a HA, or do children also need the FM system?** It's not enough to use just the HA in the classroom because the students may not understand the teacher's speech if they are far apart. Therefore, the student can miss important information.

2. **Does the FM system use batteries?** Yes, the family must recharge the battery of the transmitter every day. It lasts 10 to 12 hours, and then must be recharged. The HA consumes more battery when the FM is on. It is advisable to have extra batteries at school.

3. **Can a single transmitter be used to synchronize with more than one student's receiver?** Yes, a transmitter can be synchronized with more than one receiver. For example, a teacher can use one transmitter for two children who use HA in the same classroom.

4. **Does the FM system record the transmitter-receiver transmission?** No, the FM system only transmits the sound picked up by the microphone to the receiver attached to the HA.

5. **Does the FM system substitute the HA and/or CI?** No, it doesn't amplify sounds, it only transmits them, keeping the intensity of the teacher's voice regardless of the distance between them.

6. **How far can the student be from the teacher?** Outdoors (playground, sports ground, etc.), the range is up to 50 meters. Indoors (classroom), it is up to 30 meters.

Contact

www.derdic.com.br
www.facebook.com/derdic.pucsp/
www.youtube.com/user/Derdicpuc

(11) 5908-7983
derdicinclusao@pucsp.br

FUNDAÇÃO SÃO PAULO
mantenedora da
Pontifícia Universidade Católica de São Paulo

Derdic

PUC-SP

FUMCAD
Fundo Municipal dos Direitos
da Criança e do Adolescente

PHONAK

Rua Estado de Israel, 860 – 04022-000
Vila Mariana – São Paulo – SP
(11) 5908-7983
derdicinclusao@pucsp.br

Projeto FM

Derdic | PUC-SP

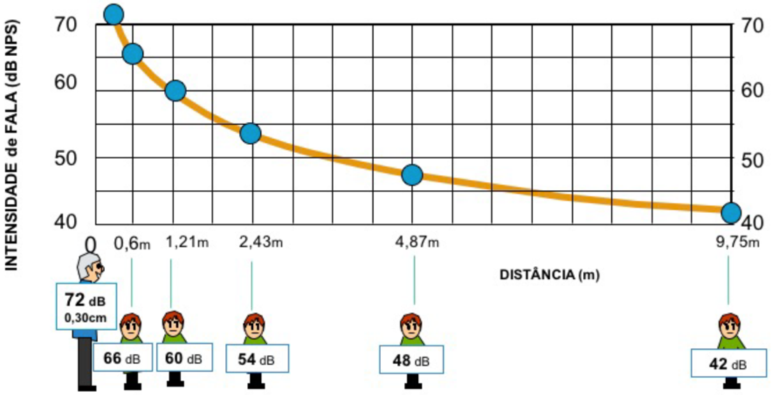

MEU NOME

Escola:

O Som e minha audição

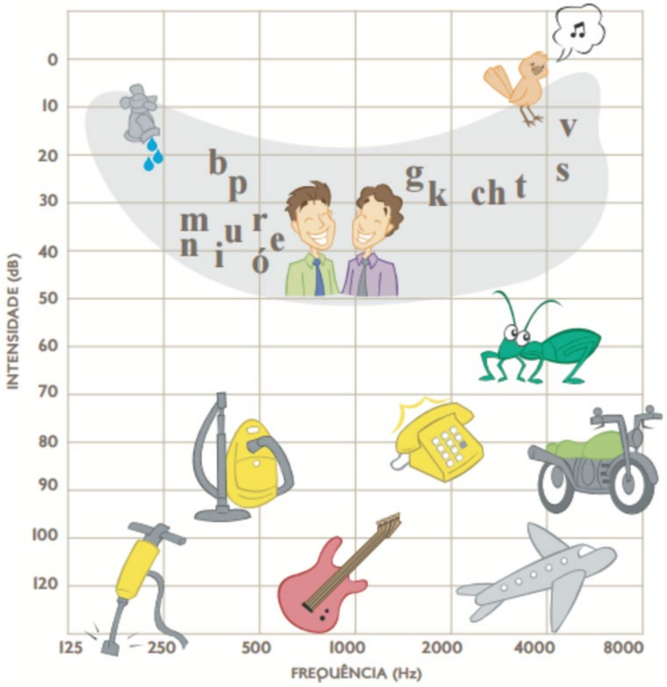

Minha perda auditiva

Tipo de perda auditiva

Orelha direita:

Orelha esquerda:

Grau da perda auditiva

Orelha direita:

Orelha esquerda:

O Som e minha audição

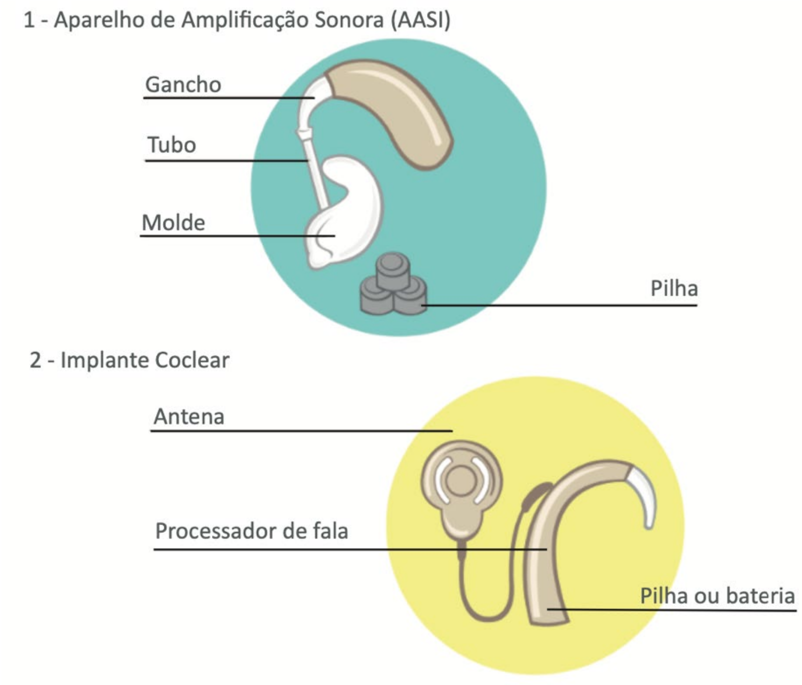

Anotações

Modelo AASI/IC
orelha direita:
Orelha esquerda:

O que meu FM faz e como funciona?

O Sistema FM é O Sistema FM é a ferramenta educacional desenvolvida crianças com deficiência auditiva usuárias de aparelho de amplificação sonora e/ou implante coclear com o objetivo de melhorar o entendimento da fala do professor, pais, terapeutas em ambientes ruidosos, diminuindo a distância entre o falante e o ouvinte, principalmente na escola.

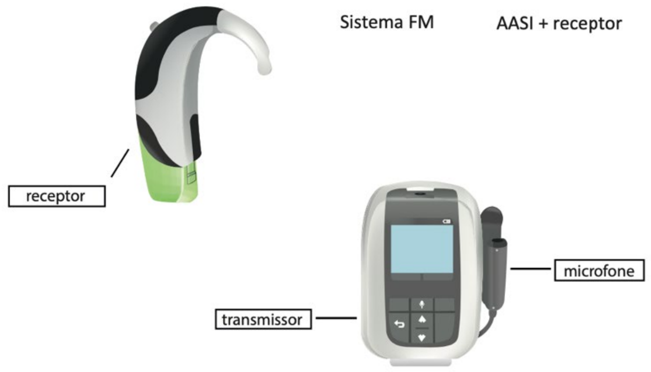

Anotações

Modelo AASI/IC
orelha direita:
Orelha esquerda:

Efeitos da Reverberação e Distância do Professor

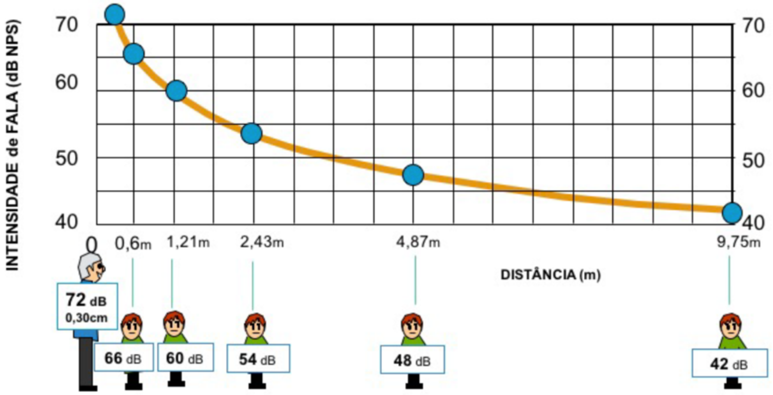

Devemos considerar todos os ambientes em que a criança vive como um local favorável de desenvolvimento de linguagem e audição, que possibilitem a interação comunicativa. A escola é um desses ambientes, pois é nela que a criança passa grande parte do seu tempo (Delgado-Pinheiro, 2009).

Os estudantes passam 45% do seu tempo na escola, envolvendo atividades que predominam a voz do professor e de seus colegas. Desse modo, é fácil concluir que a mensagem e capacidade de processá-la auditivamente é essencial para a aprendizagem escolar.

As crianças com deficiência auditiva demandam um esforço maior que seus pares ouvintes, principalmente em ambiente escolar, independente do recurso tecnológico utilizado (AASI ou IC), para conseguir compreender a mensagem transmitida (Hicks e Tharpe, 2002).

Algumas dicas para o bom uso do FM

→ O microfone deve ser preso na roupa pelo menos um palmo abaixo da boca para evitar que ruídos da respiração, da boca e de acessórios (colares, cachecol e botões) sejam amplificados.
→ Evite quedas e batidas do dispositivo
→ Quando sujo de pó de giz, deve ser limpo com um papel ou pano seco
→ Não molhe
→ Evite umidade, pois pode provocar oxidação
→ Em caso de falha de funcionamento, verifique com a família se as pilhas do aparelho estão sendo trocadas adequadamente.

Perguntas frequentes

1. **Usar aparelhos auditivos é suficiente ou as crianças precisam também de Sistema FM?** Usar somente os aparelhos auditivos não é suficiente em sala de aula, pois os estudantes podem não captar a fala do professor com o aumento da distância. Dessa forma o estudante pode perder informações importantes.

2. **O sistema FM usa bateria?** Sim, é necessário que a família carregue a bateria do transmissor todos os dias. A bateria tem duração de 10/12 horas, quando deve ser recarregada. O AASI vai consumir mais bateria/pilha quando o FM está ligado. É recomendado deixar baterias/pilhas extras na escola.

3. **Você pode usar um único transmissor para sincronizar com receptores de mais de um estudante?** Sim, um transmissor pode ser sincronizado com mais de um receptor, por exemplo uma professora pode usar um transmissor com duas crianças que utilizam aparelhos auditivos na mesma sala.

4. **O sistema FM grava a transmissão entre o transmissor e o receptor?** Não, o sistema FM apenas transmite o som captado pelo microfone para o receptor acoplado ao aparelho de amplificação sonora.

5. **O sistema FM substitui o aparelho de amplificação e/ou implante coclear?** Não, ele não amplifica o som, apenas transmite, mantendo a mesma intensidade da voz do professor independente da distância entre eles.

6. **Até que distância o estudante pode estar do professor?** Em um ambiente aberto (parquinho, quadras e etc) abrange uma área de 50 metros. Em um ambiente fechado (sala de aula) até 30 metros.

Contatos

www.derdic.com.br
www.facebook.com/derdic.pucsp/
www.youtube.com/user/Derdicpuc

(11) 5908-7983
derdicinclusao@pucsp.br

FUNDAÇÃO SÃO PAULO
mantenedora da
Pontifícia Universidade Católica de São Paulo

Derdic

PUC-SP

FUMCAD
Fundo Municipal dos Direitos da Criança e do Adolescente

PHONAK

Rua Estado de Israel, 860 – 04022-000
Vila Mariana – São Paulo – SP
(11) 5908-7983
derdicinclusao@pucsp.br
