# Supplementary material for: Adherence to Remote Microphone System use at school in children and adolescents with hearing loss
Source: Codas. 2022 Jan 12;34(3):e20200326. doi: 10.1590/2317-1782/20212020326 (PMC9769424; doi:10.1590/2317-1782/20212020326)
Supplement: Translation

# Translation: Adesão ao uso do Sistema de Microfone Remoto em estudantes com deficiência auditiva usuários de dispositivos auditivos

**Authors:** Giovana Targino Esturaro, Bruna Capalbo Youssef, Luisa Barzaghi Ficker, Tatiana Medeiros Deperon, Beatriz de Castro Andrade Mendes, Beatriz Cavalcanti de Albuquerque Caiuby Novaes

**Affiliations:** 1 Programa de Pós-graduação em Fonoaudiologia, Faculdade Pontifícia Universidade Católica de São Paulo – PUC-SP - São Paulo (SP), Brasil.

**Keywords:** Audição,Auxiliares de Audição,Tecnologia Sem Fio, Escolaridade, Pais, Perda Auditiva, Adolescente

## Abstract

**Objetivo:**

Identificar relações entre a utilização sistemática do Microfone Remoto (MR) em sala de aula de estudantes com deficiência auditiva e características das escolas e dos professores.

**Método:**

Foram analisados 120 sujeitos, entre cinco e 17 anos, com deficiência auditiva que foram adaptados MR em um Serviço de Saúde credenciada pelo Sistema Único de Saúde (SUS). Também foram sujeitos, professores de usuários de MR. Realizou-se uma análise de prontuários e no momento que os sujeitos compareceram para acompanhamento foi realizado entrevista com os pais/responsáveis para caracterizar rotina de utilização do MR na escola. Foram realizados contatos telefônicos e visitas presenciais em algumas escolas.

**Resultados:**

Quanto ao uso, observa-se que a maioria dos sujeitos utilizava o dispositivo na escola. Aqueles que não utilizavam involuntariamente e voluntariamente consistiu na minoria dos sujeitos. Houve uma similaridade no padrão do *Speech Intelligibility Index -*SII dos sujeitos que ‘usam’ e ‘não usam involuntariamente’ o MR. Houve diferença significativa entre o tipo de escola e o nível de escolaridade, a maioria dos sujeitos que frequentavam escola regular e estavam matriculados no ensino fundamental I tendem a usar mais o dispositivo.

**Conclusão:**

A maioria dos sujeitos faz uso do MR na escola. O nível educacional do estudante também foi um fator que interferiu na adesão ao uso dos MR, com maior adesão em estudantes do Ensino Fundamental I. Os dados sugerem que a articulação entre serviço de saúde e escola favorece a utilização do MR, entretanto quando essa relação é intermediada pelos pais, outros fatores acabam interferindo no uso sistemático no cotidiano da escola.

## INTRODUÇÃO

A audição é responsável pela captação de estímulos, e exerce um papel fundamental no desenvolvimento global das crianças. Um dos requisitos para a criança adquirir a linguagem oral é ser capaz de detectar, assim como, localizar, discriminar, memorizar, reconhecer e compreender os sons^(1,2)^.

Todos os ambientes em que a criança vive devem ser locais favoráveis em termos de pragmática, linguagem e audição, possibilitando não apenas a interação comunicativa, mas também um melhor aproveitamento das situações que envolvem diálogo para o seu desenvolvimento. A escola, por exemplo, é um desses ambientes e, por ser um local onde a criança passa grande parte de seu tempo, deve ser um ambiente favorável à potencialização do desenvolvimento de seus alunos^(3)^.

Além de ter um ambiente favorável, a programação e verificação do aparelho de amplificação sonora (AASI) são fundamentais para garantir a audibilidade dos sons de fala como uma das condições para o desenvolvimento de linguagem das crianças com deficiência auditiva^(4)^.

O Índice de Inteligibilidade de fala (*Speech Intelligibility Index-*SII) é uma medida objetiva realizada durante o processo de verificação dos AASI, que fornece a quantidade de informação de fala audível ao indivíduo com e sem o uso da amplificação, sendo essa uma das maneiras de quantificar a relação entre o sinal de fala e os resultados de reconhecimento de fala^(5)^.

A capacidade de processar e entender a fala dos professores é essencial para o aprendizado escolar. Estudos demonstram que, na escola, as crianças passam 45% do seu tempo envolvidas em atividades nas quais predomina a fala do professor e de seus colegas. Desta forma, ter acesso adequado ao som do professor é essencial para a aprendizagem escolar^(6,7)^. No entanto, a adesão só ocorre quando todos os envolvidos com o seu uso (profissionais da saúde, família e educadores) trabalham em parceria para garantir o uso efetivo e correto do dispositivo^(8-10)^.

Para a pessoa com deficiência auditiva, a tecnologia assistiva do Microfone Remoto (MR) é um auxílio técnico, que inclui produtos, instrumentos, equipamentos ou tecnologias adaptados, ou especialmente projetados para melhorar a funcionalidade da pessoa com deficiência auxiliando à autonomia pessoal, total ou assistida (Lei nº 5296 de 2/12/2004), dando acesso direto para a voz do interlocutor.

Neste contexto, o Microfone Remoto (MR) é uma tecnologia de transmissão sem fio que capta a voz do interlocutor (professor, terapeuta ou pais) por um microfone conectado a um transmissor que envia o sinal de fala para um receptor conectado ao aparelho de amplificação sonora individual (AASI) ou implante coclear (IC) do usuário, com o objetivo de minimizar problemas relacionados à distância, ruído e reverberação e melhorando a relação sinal/ruído nos diversos ambientes.

Com a evolução da tecnologia de Frequência Modulada (FM) para um transmissor digital, o usuário teve mais benefício devido ao fornecimento de um melhor reconhecimento de fala no ruído e pela diminuição das interferências, permitindo uma maior constância na transmissão dos sons^(11)^. Optamos por utilizar o termo Microfone Remoto (MR), que engloba todas as tecnologias de microfone remoto.

No ano de 2013, a Portaria de n^o^ 1.274/GM/MS publicada pelo Ministério da Saúde incluiu o Sistema de Frequência Modulada (FM)^(12)^ na tabela de procedimentos, medicamentos, órteses, próteses e materiais especiais (OPM) do Sistema Único de Saúde (SUS), a fim de implementar novas iniciativas e intensificar as medidas já desenvolvidas pelo governo, em benefício da pessoa com deficiência, melhorando o acesso desses cidadãos a direitos básicos, como a educação. Com isso, os usuários de AASI e/ou IC - entre cinco e 17 anos, particularmente aqueles que têm habilidades de reconhecimento de fala - puderam recorrer ao SUS para adquirir esse recurso, podendo facilitar seu aprendizado no contexto escolar.

No ano de 2020, iniciou-se a análise para ampliar o MR para indivíduos com deficiência auditiva de qualquer idade e matriculados em qualquer nível acadêmico, no âmbito do Sistema Único de Saúde – SUS, Portaria Nº 3, e 19 de fevereiro de 2020^(13,28)^.

As escolas são ambientes excessivamente ruidosos, e o ruído interfere no desempenho acadêmico dos estudantes, principalmente para aqueles com deficiência auditiva, o que tem impacto direto no esforço auditivo^(10,14)^. A reverberação em salas de aula acarreta dificuldades no entendimento da mensagem transmitida pelo interlocutor, gerando a necessidade de que haja um aumento da energia para que o sujeito possa compreender o conteúdo que está sendo ministrado. A melhor forma de diminuir a distância entre locutor e interlocutor é fazendo o uso de tecnologia assistiva, utilizando o Microfone Remoto (MR) visando a melhora da relação sinal/ruído (S/R) em sala de aula^(8,15,16)^.

A efetividade e sucesso no uso do MR depende de diversos fatores, entre eles, a parceria entre saúde e educação. Ambos precisam atuar juntos neste processo para beneficiar o estudante, no que diz respeito à criança com deficiência auditiva que se comunica oralmente, é preciso garantir o acesso à percepção dos sons da fala e ao conteúdo pedagógico^(17,18)^. Contudo, no decorrer desta relação, algumas dificuldades surgem, tais como: excesso de burocracia, falta de recursos humanos e falta de tempo, o que dificulta a parceria entre escola e saúde^(19,20)^.

Em relação ao nível de escolaridade é demonstrado que os que estão no Ensino Fundamental I (EF-I) utilizam mais o MR do que os que frequentam o fundamental II e ensino médio. Neste mesmo estudo, observou-se que os sujeitos que não usavam voluntariamente o equipamento tinham uma boa audibilidade (acima de 60%) em situação de silêncio^(21)^.

Um estudo que analisou 185 prontuários de adolescentes que foram adaptados com MR nos anos de 2013 a 2016 relata que dos sujeitos que não usavam o dispositivo, alguns ficaram envergonhados por usar o dispositivo (40%), outros devido à quebra do aparelho de amplificação sonora individual (AASI) ou do MR (20%), falta de apoio do professor ou estudantes não sentem a necessidade de usar (27%), dificuldade no manuseio e rejeição ao AASI (10%)^(22)^.

O serviço de saúde auditiva ou Centro Especializado em Reabilitação (CER) deve, além de fazer adequadas adaptações dos MR para o aparelho de amplificação sonora da criança, deve também oferecer auxílio necessário e rápido para a solução de problemas e adaptação do dispositivo por parte da escola. Desta forma, o objetivo deste trabalho foi identificar relações entre a utilização sistemática do Microfone Remoto em sala de aula e características dos estudantes, das escolas e dos professores.

## MÉTODO

Trata-se de estudo exploratório de caráter qualitativo e quantitativo, realizado com professores de estudantes com deficiência auditiva que utilizam o MR no cotidiano escolar. Este estudo foi submetido ao Comite de Ética em Pesquisa da Pontifícia Universidade Católica de São Paulo e Plataforma Brasil, sendo aprovado com o parecer número 1.110.125 (CAEE – 45415514.1.0000.5482). Todos os responsáveis dos sujeitos assinaram o termo de consentimento livre e esclarecido.

Foram analisados 120, na Tabela 1 podemos ver a caracterização dos sujeitos deste estudo com deficiência auditiva do tipo neurossensorial de grau leve a profundo que foram adaptados com MR e realizam acompanhamento audiológico em um Serviço de Saúde credenciada pelo Sistema Único de Saúde (SUS) como Centro Especializado em Reabilitação II – Auditivo e Intelectual, na cidade de São Paulo durante o período de janeiro a dezembro de 2017. Também foram sujeitos deste estudo, professores de usuários de MR. O critério de inclusão da pesquisa se baseia nos critérios descritos na Portaria do FM 1.274.

**Tabela 1:** Caracterização dos sujeitos

Categorização do Sujeitos					
**Sexo**		Feminino	Masculino		
	N	59	61		
	%	49%	51%		
**Nível Socioeconomico**		A+B1	B2+C1	C2+D+E	
	N	8	67	45	
	%	7%	56%	38%	
**Nível Educacional**		Fundamental I	Fundamental II	Ensino Médio	
	N	68	41	11	
	%	57%	34%	9%	
**Grau da Perda auditiva**		Normal/Leve	Moderada	Severa	Profunda
	OD	21	38	35	26
	OE	12	40	36	32

Legenda: OD- orelha direira; OE- orelha esquerda

Antes da avaliação, realizou-se uma análise de prontuários para a obtenção de informações como sexo, idade, limiares audiométricos de 500 Hz a 4 kHz de ambas as orelhas, valor do Índice de Inteligibilidade de Fala – SII 65dB da melhor orelha, região de moradia, nível de escolaridade do estudante (Ensino Fundamental I - EF-I, Ensino Fundamental II - EF-II, Ensino Médio e Supletivo), classificação socioeconômica, nível de escolaridade dos pais ou responsáveis, e o tipo de escola que o estudante frequentava (regular, regular-intérprete e especial - LIBRAS).

No retorno ao serviço, para acompanhamento (momento do retorno semestral ou anual na instituição para realizar novos exames audiológicos e verificações dos AASI), eram realizados:

1. Entrevista com os pais/responsáveis com objetivo de verificar questões do MR e o seu uso na escola;
2. Aplicação do questionário de classificação socioeconômica das famílias^(23)^;
3. Verificação e média de horas diárias de uso do AASI;
4. Verificação de funcionamento do MR;
5. Classificação quanto ao uso do MR:

Usa

- No caso em que usa regularmente na escola (usa mais de 1 hora e meia por dia na escola)

Não usa involuntariamente/ impedido de usar

- MR está no conserto;
- MR foi perdido ou/roubado;
- Professor não quer usar o MR na escola.

Não usa voluntariamente

- Devolveu o MR;
- Escola especial (Libras);
- Estudante não quer usar o MR na escola.

1. Entrega de material para os responsáveis encaminharem para a equipe pedagógica da escola, contendo: instruções para o bom uso do equipamento, SIFTER Brasileiro - Instrumento de Identificação do Risco Educacional em Estudantes do Ensino Fundamental e Médio, carta de apresentação da pesquisa; termo de consentimento livre e esclarecido.

### Contato telefônico com todas as escolas

Além do material enviado pelas famílias, foram realizados contatos telefônicos e visitas presenciais em algumas escolas, já que é protocolo da instituição oferecer uma visita às escolas para explicação e orientação sobre os MR, mesmo quando os pais não trazem essa demanda. Foram priorizadas as escolas nas quais os pais e as crianças trouxeram a necessidade de visita, ou por solicitação da escola.

Todas as escolas estavam dentro do município da cidade de São Paulo. Para a localização do endereço e responsável pelas escolas, foram realizados os seguintes procedimentos:

1. Busca no *Google* com o nome da escola para localizar o telefone e endereço da escola, ou informações trazidas pelos pais;
2. Contato telefônico com as escolas dos estudantes da pesquisa para conversa com o professor, coordenador, professor da sala de apoio, assistente de direção e/ou intérprete responsável pelo estudante; eram convidados a participar da visita escolar quem estivesse disponível no momento;
3. Agendamento da visita conforme disponibilidade da escola. Não foram realizadas visitas nos casos em que o MR estava quebrado, ou aguardando receptores compatíveis (não uso involuntário).

### Visita escolar

- Agenda da visita na escola do estudante via telefone com o profissional disponível responsável pelo estudante.

Roteiro da visita escolar:

1. Apresentação do MR para explicação do benefício de como utilizar o dispositivo e manusear o equipamento junto com a equipe pedagógica presente na reunião;
2. Apresentação de um vídeo com a demonstração do benefício do equipamento, mostrando a diferença de como a criança escuta com o AASI e com o AASI+MR e cuidados com o equipamento;
3. Demonstração do MR com um fone de ouvido para que os professores/coordenadores tivessem a sensação real de estarem utilizando o MR e que pudessem se sentir como os alunos escutam em sala de aula;
4. Caso a criança estivesse na escola e pudesse ser interrompida, era solicitada uma visita à sala de aula do aluno para demonstrar o uso do MR;
5. Entregue para a escola o livreto explicativo (Anexo 1) e o questionário SIFTER Brasileiro - Instrumento de Identificação do Risco Educacional em Estudantes do Ensino Fundamental e Médio^(24)^.

Após a visita à escola, agendamos novamente as famílias (ou elas já tinham um retorno previamente agendado) para um retorno quanto à visita e às novas orientações.

### Análise dos dados

Foi realizada a análise de relações entre características audiológicas e demográficas dos sujeitos e das escolas, relacionando-as à adesão ao uso do MR. Além disso, foi realizada a análise qualitativa a partir de visitas às escolas para orientação e obtenção dos relatos quanto às dificuldades e/ou benefícios do uso do MR pelo aluno.

Os dados foram digitados em planilha de dados e analisados no *software Statistical Package for the Social Sciences* (SPSS) versão 22.0 para Windows. Para a comparação das variáveis qualitativas, decidiu-se pela utilização de testes não paramétricos, especificamente, o teste de *Kruskal-Wallis* para comparações de duas ou mais categorias e, no caso da existência de diferença significante, o teste de *U de Mann-Whitney* para as comparações par a par entre os grupos.

## RESULTADOS

Cento e vinte sujeitos que foram adaptados com o MR compareceram para o acompanhamento na instituição no ano em que foi realizada a coleta. Destes, 54% (n=65) eram do gênero feminino e 46% (n=55) do gênero masculino. Quanto à classificação socioeconômica, 63% (n=76) eram de classe C, 28% (n=34) de classe B, 8% (n=9) de classe D e uma família (1%) era de classe A.

Quanto ao uso do dispositivo, foi observado que 54% (n=65) utilizavam o dispositivo na escola; 22% (n=26) não utilizavam involuntariamente (por perda, roubo, AASI ou MR no conserto ou incompatibilidade do AASI com o receptor e/ou professor não quer usar); 24% (n=29) não utilizava voluntariamente (por terem devolvido o equipamento alegando não perceber benefício, não utilizavam linguagem oral como principal forma de comunicação, utilizavam LIBRAS como principal modo de comunicação e frequentam escola especial, onde os professores ministram as aulas nesta língua; e/ou o estudante não queria usar o dispositivo na escola).

Em relação à perda auditiva, foi utilizado o SII – Índice de Inteligibilidade de Fala para definir a variável grau de perda. A Figura 1 demonstra a distribuição quanto ao uso do MR e o SII do sujeito. Pode-se observar na Figura 1 que os sujeitos que ‘usam’ e ‘não usam involuntariamente’ uma grande variabilidade no valor de SII de 65 dB. Dentre os que ‘não usam voluntariamente’ há uma tendência do SII ser de maior valor. Essa diferença foi significativa (p menor 0,05).

**Figura 1:** *Box-plots* da distribuição dos sujeitos quanto ao uso do SMR - usando ou impedido de usar, agrupados e - não usando, em relação ao SII – Índice de inteligibilidade de fala (n=120)

Nota-se, que a menor média e a menor mediana de uso diário do AASI foram observadas entre aqueles que não usam voluntariamente. As distribuições dessa variável estão representadas na Figura 2.

**Figura 2:** *Box-plots* da mediana de uso diário do AASI em cada categoria de uso do microfone

Em relação ao tipo de escola, 86% (n=103) frequentavam escola regular, 7% (n=8) frequentavam escola regular com intérprete de LIBRAS e 7% (n=9) frequentavam escola especial, com ensino exclusivo em LIBRAS. Dentre os 120 sujeitos, 57% (n=68) estavam matriculados no EF-I, 34% (n=41) no EF-II e 9% (n=11) no ensino médio.

Quando comparados os níveis de escolaridade com o uso do MR, podemos observar altos níveis de significância; no comparativo dos três níveis de escolaridade a significância foi p=0,028. Quando comparado apenas o nível do EF-I e Ensino médio, a significância foi de p=0,008.

Em análise comparativa quanto ao nível de escolaridade, observou-se que, dos sujeitos matriculados no EF-I, 62% utilizavam o MR, sendo esse o nível de escolaridade com maior adesão ao uso do dispositivo, seguido pelo ensino médio (55%) e EF-II (41%). Deve ser ressaltado que só havia 11 sujeitos no ensino médio, o que dificulta a comparação (Figura 3).

**Figura 3:** Distribuição dos sujeitos relacionando a situação de uso do SMR e o nível educacional do estudante (n=120)

A distribuição de uso do MR dos sujeitos do EF-I (n=68) e do EF-II (n=41), quando comparadas de forma isolada entre si, apresentam algumas diferenças quanto ao índice de sujeitos em cada categoria analisada, demonstradas a seguir.

O EF-I apresenta números superiores em relação ao EF-II no número de sujeitos ‘usando’ (diferença do n=25) e que ‘não usa involuntariamente’ (diferença de n= 9).

O EF-II supera o EF-I somente quanto ao número de sujeitos que ‘não usa voluntariamente’ (diferença de n=7).

Em outra perspectiva, os sujeitos que estavam usando o MR na escola foram agrupados aos que não estavam usando devido a fatores alheios à sua vontade (não usando involuntariamente). Com essa distribuição, fica mais evidente a maior dificuldade de adesão ao uso no grupo do EF-II (Figura 3 e Figura 4).

**Figura 4:** Distribuição dos estudantes relacionando a situação de uso do SMR - usando ou impedido de usar, e - não usando, em relação ao nível educacional do estudante (n=120)

Fazia parte do contato com a escola o encaminhamento do questionário SIFTER Brasileiro, tanto via família, quanto levado no momento da visita escolar para entregar à equipe pedagógica que recebia os profissionais de saúde. Dos 120 questionários encaminhados, retornaram apenas seis (5%). Devido à baixa adesão de respostas, não foi realizada uma análise das respostas desse instrumento.

Em um recorte qualitativo a fim de aprimorar o processo de adaptação do dispositivo nas escolas e atender à demanda de familiares, foi realizado o contato telefônico com as escolas, e o agendamento de visitas presenciais em 28 escolas, como podemos observar no mapa abaixo (Figura 5).

**Figura 5:** Distribuição dos sujeitos das escolas visitadas e dos contatos telefônicos realizados (n=120)

Catorze visitas foram agendadas por demandas trazidas pelos familiares dos usuários com o objetivo de dar uma melhor orientação para a equipe pedagógica, e outras 14 visitas foram realizadas seguindo o protocolo da instituição, quando havia concordância das escolas.

Em relação às escolas visitadas, em 75% (n=21) as crianças estavam utilizando o MR em sala de aula; em 14% (n=4) o aluno “não se adaptou ao uso do MR”, segundo a escola; em 7% (n=2) os alunos estavam com AASI/MR no conserto e um (3%) era uma escola de ensino especial (LIBRAS). A disponibilidade em receber a equipe do serviço para orientação foi provavelmente um fator determinante nessa distribuição. Nas escolas onde os alunos usavam com regularidade, parece ter havido maior interesse em receber orientações.

Em 54% das escolas visitadas (n=15), participaram da reunião professores e coordenadores; em 29% (n=8) apenas o coordenador participou, e se prontificou a passar o conteúdo da visita aos professores em outro momento; em 21% (n=6) apenas os professores participaram.

Considerando que 23 das 28 escolas que concordaram com a visita eram escolas onde os sujeitos estavam usando o MR em sala de aula, os coordenadores estiveram presentes em 91% das visitas e em 56% das mesmas estiveram o coordenador e o professor.

## DISCUSSÃO

Participaram do estudo 120 sujeitos que foram adaptados com MR no ano de 2017 que fizeram parte do projeto do Termo de Convênio, firmado entre a o Centro Especializado em Reabilitação – deficiência auditiva e a Prefeitura do município de São Paulo, por meio da Secretaria de Participação e Parceria/Conselho Municipal dos Direitos da Criança e do Adolescente (CMDCA).

A adesão ao MR é muito mais complexa do que simplesmente entregar o dispositivo e esperar que o sujeito tenha sucesso na sua utilização sem que ocorra articulação entre familiares, professores e profissionais da saúde^(17,18)^.

Podemos observar que 54% (n=65) de estudantes estão utilizando o dispositivo na escola, 22% (n=26) não utilizavam involuntariamente; e 24% (n=29) não utilizavam voluntariamente. De acordo com os autores^(10,20)^, isso pode estar relacionado a diversos fatores, como pouca informação dada ao professor sobre como utilizar o equipamento, além de desconhecimento sobre a perda auditiva do estudante; em outros casos, o próprio estudante se negava a utilizar o dispositivo, alegando vergonha dos colegas.

Neste estudo, fizemos uma relação do uso do equipamento com o SII - Índice de Inteligibilidade de Fala. Os sujeitos que ‘usam’ e ‘não usam involuntariamente’ têm uma similaridade no padrão de SII e os que ‘não usam voluntariamente’, o SII tem uma tendência a ser superior, podendo ser justificado pelo fato de estudantes não terem uma percepção da diferença com e sem MR no momento inicial e a família não compreender a real necessidade do dispositivo e não insistir na utilização no mesmo^(10,21)^. Os sujeitos que não usavam voluntariamente o equipamento tinham uma boa audibilidade (acima de 60%) em situação de silêncio, o que pode justificar a baixa adesão ao MR nos momentos iniciais da adaptação, pois eles alegam escutar bem apenas com o AASI ou IC^(21)^.

A portaria do Ministério da Saúde não prevê a reposição por perda, roubo ou defeito do equipamento; na amostra deste estudo, 22% dos sujeitos ‘não utilizam involuntariamente’ o dispositivo devido à perda, roubo, AASI ou MR no conserto ou incompatibilidade do AASI com o receptor do MR e/ou professor se recusou a utilizar, condizente com a literatura. Um outro estudo com a população que recebeu o MR entre os anos de 2013 e 2016, 16% estavam com o equipamento quebrado^(22)^.

Muitos sujeitos deixaram de utilizar o MR, por fatores que não dependiam deles e foram, desta forma, privados do benefício deste equipamento. Quando agrupamos os usuários do MR com aqueles que usariam, porém estão impedidos de usar, essa diferença deixa mais evidente o quanto aumentaria o número de usuários utilizando o equipamento na escola. Estes usuários não seriam privados do uso do MR se fossem colocadas em prática as políticas de reposição/ substituição destes dispositivos conforme apresentado na portaria 1.274 e de acordo com o relatório nº 58 da CONITEC, o impacto orçamentário decorrente da incorporação dessa tecnologia assistiva que levou em consideração o público-alvo originariamente tido como passível desse tipo de intervenção. A disponibilização dos sistemas de MR pelo SUS representa um grande avanço para a reabilitação de crianças com deficiência auditiva. No entanto, ainda está abaixo do recomendado pelo Ministério da Saúde, com distribuição desigual nas regiões do país e observou-se uma diminuição na disponibilização destes dispositivos nos últimos anos^(25)^. Alguns estudos^(25,26)^ argumentam que tais tecnologias possuem uma vida útil de quatro anos e demandam substituições periódicas. No entanto, passados dois anos da referida previsão de duplicação do quantitativo das concessões, não houve a substituição dos equipamentos dos Sistemas FM, nem a revisão da tecnologia indicada.

O nível educacional do estudante também foi um fator que interferiu na adesão ao uso dos MR; 57% (n=68) dos sujeitos estão matriculados no EF-I e estes são os que mais utilizam o MR (62%). Isto está de acordo com os testes estatísticos realizados, tendo como nível de significância p=0,028 quando foram comparados os níveis de escolaridade (EF I, EF II e Ensino médio) com o uso do dispositivo. Pode-se observar que o uso no ensino fundamental I (EF-I) é maior mesmo quando agrupamos os que ‘não usavam involuntariamente’ e, quando comparado o nível de escolaridade (EF I e EM) com o uso do MR, também resultou em significância de p=0,008. Essa diferença encontrada sugere que o fato dos estudantes do EF I terem apenas um professor em sala de aula facilita o uso do dispositivo eletrônico, as orientações e a adesão ao uso no cotidiano escolar, o que também foi observado pela autora^(21)^.

A literatura demonstra que a articulação entre saúde e escola ainda apresenta dificuldade^(19)^. Neste estudo, pode-se verificar que o contato com a escola não foi um processo simples; sendo necessário telefonar diversas vezes para obter sucesso; as longas distâncias percorridas pelos profissionais para chegarem à escola é outro fator que interfere na adesão^(20,27)^.

Isto também fica evidente na baixa adesão de resposta ao questionário, em que apenas 5% dos professores responderam ao SIFTER e a principal justificativa dada foi a falta de tempo para preencher e enviar o questionário. Segundo a autora^(19)^, a falta de tempo relatada também foi um fator que dificultou a aproximação entre as áreas da saúde e educação.

Quando analisamos as crianças que não utilizam o MR e as relacionamos aos participantes das reuniões durante as visitas à escola, identificamos que foram o professor da sala de apoio e do atendimento educacional especializado (AEE). Vale ressaltar que estes profissionais passam apenas algumas horas semanais com as crianças, mostrando que a não participação do professor principal e do coordenador pedagógico durante esses encontros pode interferir negativamente no uso do MR.

Diferente de quando são analisados os sujeitos que utilizavam o MR; a maioria (56%) teve a participação na reunião dos professores e coordenadores. Pode-se concluir que toda a equipe reunida durante esses encontros possibilita uma maior adesão ao uso do MR, uma vez que o uso não é individual do estudante e sim coletivo da escola, família e sistema de saúde.

Para o sucesso no uso do MR é essencial a articulação entre saúde, família e educação, considerando que a relação entre o serviço de saúde e a escola parece não ocorrer de modo sistemático, ficando na dependência de contatos informais entre as equipes. Parece ser necessário rever a portaria do Ministério da Saúde para reposição do dispositivo por perda ou roubo, incompatibilidade de receptores no caso de troca de AASI, e manutenção no caso de defeito no dispositivo.

A continuidade de pesquisas nessa mesma temática é fundamental para ampliar o olhar para a inclusão da criança oralizada no ambiente escolar e a criação de mais protocolos que favoreçam a adaptação do MR na escola. Da mesma forma, a parceria entre saúde e educação deve ser fortalecida para beneficiar a criança com deficiência auditiva.

## CONCLUSÃO

Foi possível concluir que:

- A maioria dos sujeitos fez uso do MR na escola;
- Perda, roubo e defeito foram fatores presentes dentre as razões do ‘não uso involuntário’ do dispositivo;
- O nível educacional do estudante também foi um fator que interferiu na adesão ao uso dos MR; os alunos matriculados no EF-I são os que mais utilizam os MR;
- A dificuldade em acessar o professor é um fator que contribui para a não adesão ao uso do dispositivo, pois nem sempre foi possível dar as orientações necessárias para seu uso consistente e adequado.

## Material Suplementar

Este artigo acompanha material suplementar.

**Anexo 1:** O Som e minha audição

Este material está disponível como parte da versão online do artigo na página: https://www.scielo.br/j/codas
